# NMR Characterization of Self-Association Domains Promoted by Interactions with LC8 Hub Protein

**DOI:** 10.5936/csbj.201402003

**Published:** 2014-02-25

**Authors:** Elisar Barbar, Afua Nyarko

**Affiliations:** a Department of Biochemistry and Biophysics, Oregon State University, Corvallis, Oregon 97331, United States

## Abstract

Most proteins in interaction networks have a small number of partners, while a few, called hubs, participate in a large number of interactions and play a central role in cell homeostasis. One highly conserved hub is a protein called LC8 that was originally identified as an essential component of the multi-subunit complex dynein but later shown to be also critical in multiple protein complexes in diverse systems. What is intriguing about this hub protein is that it does not passively bind its various partners but emerging evidence suggests that LC8 acts as a dimerization engine that promotes self-association and/or higher order organization of its primarily disordered monomeric partners. This structural organization process does not require ATP but is triggered by long-range allosteric regulation initiated by LC8 binding a pair of disordered chains forming a bivalent or polybivalent scaffold. This review focuses on the role of LC8 in promoting self-association of two of its binding partners, a dynein intermediate chain and a non dynein protein called Swallow.

## Introduction

LC8 is a highly conserved homodimeric protein that assembles in the molecular motor dynein by binding dynein intermediate chain IC [1–3]. LC8 also interacts with diverse proteins, some of which are associated with active transport within the cell. For example with Egalitarian, a protein essential for establishing and maintaining polarity during oogenesis and embryogenesis in *Drosophila* [4], mutations that disrupt its binding to LC8 result in failure of accumulation of oocyte-specific markers. Another interaction is with Dazl which is required for mRNA localization in male germ cell development in mammals, and whose distribution is microtubule-dependent [5]. With Swallow, LC8 binding is required for dynein-dependent transport of bicoid mRNA to the oocyte anterior cortex [6]. These processes depend on the association with a microtubule-based motor suggesting a role for LC8 in active transport along dynein and fostering the hypothesis that LC8 acts as a dynein cargo adaptor [6, 7] linking proteins to dynein for transport along microtubules. However, crystal structures of LC8 bound to Swallow and IC peptides show that both partners bind the same symmetrical grooves at the LC8 dimer interface [1, 8, 9]. Moreover, LC8 in both cases binds two chains of the *same* protein [2, 10] arguing against the one groove one peptide model [11]; therefore, LC8 cannot simultaneously bind to dynein IC and non dynein partners, suggesting that all identified LC8 partners cannot be cargo transported by dynein by binding LC8 as commonly thought. Rather, nature chose one protein to do the same function in dynein IC as in all these other systems and as such LC8 is a hub protein with a common mode of action in various systems [12, 13]. Table 1 lists the LC8 binding partners for which, there is either experimental evidence of binding or a clear recognition sequence. Most binding partners were initially identified by yeast two-hybrid screens, and subsequently verified by GST pull-down assays. These binding partners have diverse roles in the cell, and varying subcellular localization including the cell nucleus, and for many, their activity is regulated by LC8 binding. Other binding partners identified by pepscan/proteomics or other biochemical analyses not listed in Table 1, include: Kidney ischemia development protein (Kid-1), Protein 4, MORC family CW-type zinc finger protein 3 (MORC3), Phototropin, DNA methyltransferase 3A (DNMT3A), Spindle and centriole-associated protein 1 (Spice1), Echinoderm microtubule-associated protein-like 3 (EML3), Human papillomavirus type 8 protein E4, Heatshock cognate protein (Hsc73), microtubule-associated protein 4 (MAP4), Microtubule affinity regulating kinase 3 (Mark3), Serine/threonine-protein kinase Nek9, Guanine nucleotide-binding protein subunit beta-2-like 1 (RACK1), Flagellar radial spoke protein 3 (RSP3), and African swine fever virus (p54) [14–21].

**Table 1:** LC8 binding proteins and their functional role.

Binding Partner	Function	Functional role of LC8
Adenovirus protease (Adenain)	Cleaves viral precursor proteins	Subcellular localization [39]
ASCIZ	DNA damage response/developmental transcription factor	Subcellular localization [13, 40–42]
Bassoon	Organization of the cytomatrix at nerve terminals	Regulates axonal trafficking and synaptic levels of Bassoon [43]
BCL-2-interacting mediator (Bim)	Apoptosis	Inhibits proapoptotic activity [44]
Chica	Mitotic spindle adaptor protein	Required for spindle orientation and asymmetric cortical localization of dynein [45]
Cip-interacting zinc finger protein 1 (Ciz1).	May regulate subcellular location of CIP/WAF1	Regulates cell cycle progression of cancer cells [46]
Daz1	Male germ cell development	Subcellular localization [5]
Dynein intermediate chain (IC)	Subunit of the cytoplasmic dynein motor complex	Promotes IC self-association and stability [3, 22, 24]
Egalitarian	mRNA localization	Subcellular localization [4]
Estrogen receptor (ESR1)	Nuclear hormone receptor involved in regulation of gene expression	facilitates estrogen-induced ER transactivation and anchorage-independent growth of breast cancer cells [47]
Gephyrin	Postsynaptic scaffolding protein	Subcellular localization [48]
Guanylate kinase-associated protein (GKAP)	Trafficking of the postsynaptic densitiy-95 complex	Subcellular localization [49]
Ionotropic glutamate receptor N-methyl-D-aspartate-like 1A (GRINL1A)	Subunit of DNA-directed RNA polymerase II	Subcellular localization?^50^
Kibra	Transcriptional coactivator of estrogen receptor 1 (ESR1)	Essential for estrogen receptor transactivation in breast cancer cells [51]
Lyssavirus phosphoprotein	Viral infection	Role in mechanism of virus-induced pathogenesis [52]
Myosin Va	Transport of cellular cargo along actin filaments	Promotes assembly of the coiled-coil domain [35–37]
Neuronal nitric oxide synthase (nNos)	Catalyzes production of nitric oxide	Inhibitor [53]
Nuclear respiratory factor 1 (NRF-1)	Transcription regulation	Subcellular localization [54]
Nup159	Nuclear transport	Dimerizes and stabilizes the Nup82-Nsp1-Nup159 nucleoporin [55, 56]
p21-activated kinase -1 (Pak1)	Nuclear transport/cancer development	Proposed to modulate nuclear localization and/or activity [57]
p53 BP1	DNA repair	Subcellular localization [11]
Pilin	Required for virulence by bacterial pathogens	Possible role in host defense mechanism [58]
PTH mRNA	Calcium homeostasis	Mediates interaction with microtubules in the parathyroid gland [59]
Rabies virus P protein	Viral transcription and replication.	Subcellular localization [60]
RasGRP3	An exchange factor for Ras-like small GTPases	Subcellular localization [61]
Swallow	Localization of bicoid mRNA	Promotes self-association of the coiled-coil domain [6, 10]
Syntaphilin.	Controls mobility of axonal mitochondria through static interaction with microtubules.	Stabilizes helical coiled-coil domain within the microtubule binding region that could enhance syntaphilin-microtubule docking interactions [34].
Translocate promoter region (TPR)	Nucleoporin, role in cell division and mitotic spindle checkpoint signaling	Proper chromosome segregation [62]
Trichorhinophalangeal syndrome I (TRPS1)	Repressor of GATA-regulated genes [63]	Suppresses transcriptional repression activity [63]

We propose that a common role for LC8 in these systems is to bind partially disordered protein partners that have propensity to dimerize, and to promote their dimerization and/or higher order structural organization. The challenge in testing this hypothesis is in identifying methods suitable for characterization of large disordered proteins with heterogeneous dynamics that change not only in structure upon binding LC8 but also in their self-association. This review highlights our successes of combining several low resolution spectroscopy techniques, thermodynamics, and high resolution NMR spectroscopy in characterization not only of the initial and final structures but also of the process of complex formation.

## LC8-promoted IC self-association

The first observation that suggests structural changes in IC upon LC8 binding is the CD-detected increase in helical structure. Spectra of primarily disordered free IC and LC8-bound IC were compared after subtracting the LC8 contribution from the LC8-bound IC spectrum [3]. The latter is justified considering that structures of free and IC-bound LC8 are virtually identical [8]. The increase in helical structure was assigned to a segment C-terminal of the LC8 site, both by CD experiments performed on IC constructs of different lengths, and by limited proteolysis followed by mass spectrometry to identify fragments of IC that are protected upon LC8 binding [22].

**Figure 1:**
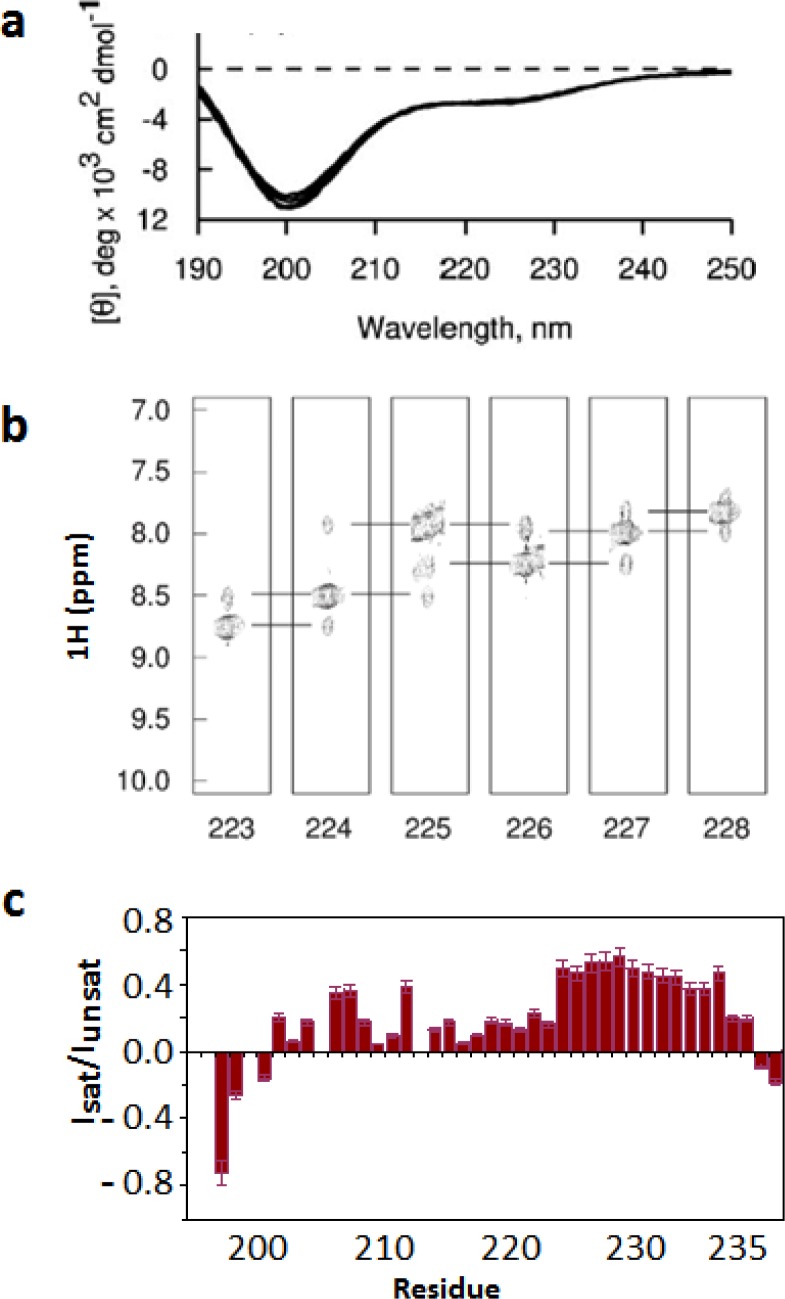
Identification of residual structure in IC. (a) Temperature-dependent far UV CD spectra of IC198-237 in the 5–25 °C temperature range, indicate that this segment is predominantly unfolded. (b) Strip plots from 3D ^1^H-^15^N NOESY-HSQC experiments recorded at 5 °C showing sequential amide-amide NOE connectivities (horizontal lines). A complete set of strong amide-amide NOEs for residues 223–228 is only observed at 5 °C suggesting formation of a nascent helix at this temperature. (c) Steady-state heteronuclear NOEs recorded at 5 °C indicate ordered structure for this segment at the nanosecond-picosecond timescale with the highest order corresponding to residues 222-232 (high positive heteronuclear NOEs). NOE values (y-axis) were determined as the ratios of the peak intensities measured from spectra recorded with and without proton saturation. Figures were adapted from [2].

The helical structure is only stabilized in the presence of LC8 as no evidence of a CD-detected helical structure is observed in free IC (Figure 1a). The specific residues involved in self association were originally mapped to 222-228 due to their propensity to form helical structure in the absence of LC8 as observed by sequential amide-amide NOEs in 3D edited ^15^N NOESY spectra (Figure 1) and their ^13^Cα and ^13^Cβ secondary chemical shifts [2]. Self-association of IC in this region is also supported by co-immunoprecipitation experiments which show a minor population of dimer (or higher oligomer) in IC constructs that include residues 200-250 [23]. NMR dynamics measurements of this domain show evidence of residual structure at the nanosecond-picosecond timescale with the highest order corresponding to residues 222-232 (high positive heteronuclear NOEs, Figure 1c).

**Figure 2:**
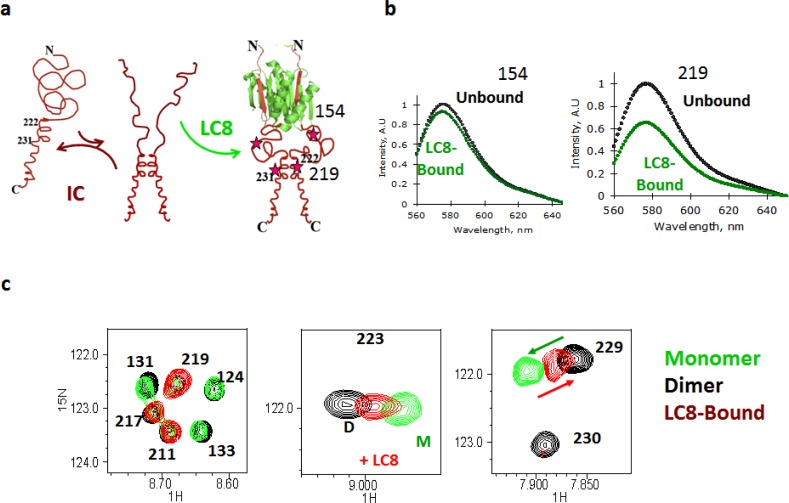
Identification of self-association domain in IC promoted by LC8 binding. (a) A model of LC8-induced IC self-association. Primarily monomeric IC (red) is in equilibrium with a small population of dimer. The latter binds LC8 (green) with a higher affinity resulting in a complex of dimer IC and LC8. Fluorescence labels inserted at sequence positions 154 or 219 are shown in red stars. (b) Fluorescence quenching upon LC8 binding is only observed when the label is inserted at position 219, confirming that these two chains in the presence of LC8 become in close proximity around residue 219 (green spectrum). For comparison, no quenching is observed when the label is placed at position 154 indicating that the IC segment around 154 remains disordered and is not part of the self-association domain. Experiments were done on a construct of IC corresponding to residues 92-260, IC92-260, that includes the LC8 recognition sequence and the putative self-association domain. (c) ^1^H-^15^N HSQC overlay spectra for a domain of IC that is fully monomeric (green), dimeric (black) and LC8 added to the monomeric form (red). The spectrum on the left shows no change in chemical shifts between dimer and monomer (perfect overlay of green and black) for these specific residues indicating that they are not at the IC dimer interface. Peaks 124, 131, and 133 disappear in the LC8-bound state confirming binding. The spectra in the center and right show chemical shift differences between monomer and dimer for residues 223 and 229, with a shift towards dimer in the LC8-bound state (red arrow). Experiments were done on a construct of IC containing residues 123-260 with linker residues 143-198 deleted. Figures b and c were adapted from [24].

LC8 binding coupled to IC self-association distant from LC8 site was confirmed by fluorescence quenching which involved inserting fluorescence labels in the monomeric free IC at different sites, one site at a time, followed by adding LC8 and monitoring the effect on the fluorescence signal intensity [24]. If a probe is present within the self-association domain, the expectation is that there will be significant self-quenching upon LC8 binding, while no change in intensity is expected when the probes are placed outside the self-association domain (Figure 2). The absence of quenching with residue 154 and the observed quenching with residue 219 clearly show that the self-association domain is separated from the 126-138 LC8 recognition sequence by a long disordered linker that ends around residue 219.

NMR studies to identify the exact boundaries of the self association domain without the destabilizing effects of the fluorescence probes were only made possible after a break-through in sample preparation methods that allowed production of a long chain of IC that includes both the LC8 site and the self-association site at conditions suitable for NMR. This involved design of a construct with a shorter linker, the use of fresh samples for every titration, and the discovery that a small amount of denaturant, 0.1 M GdnCl, is sufficient to break up the dimer to form a fully monomeric protein at NMR concentrations. Figure 2c shows spectral overlays of fully monomeric IC (green), dimeric IC (black) and LC8 bound to a monomeric IC (red). Binding of IC to LC8 in 0.1 M GdnCl is confirmed by the observation that the same peaks that broaden upon LC8 binding also broaden in the presence of GdnCl. Only the peaks that shift are assigned to those that change conformation from monomer to dimer. The rest of the peaks either do not change or disappear. This method unambiguously assigns the self-association sequence to correspond to residues 220-232 [24].

Similar fluorescence and NMR studies were performed on IC bound to Roadblock (LC7) [24], another dimeric dynein light chain that, interestingly, binds IC at a site that includes the self-association domain promoted by LC8 [25]. Both the crystal structure and increased fluorescence intensity indicate that any IC-IC contacts in the vicinity of residue 219 are not likely when LC7 is bound. Therefore, while LC8 and to a lesser extent the LC8-like light chain Tctex1, which binds at residues 110-123, promote IC self-association at residues 220-232, LC7 binds at residues 220-258 and forces unpacking of the self-association domain. Binding studies with the self-associated bivalent IC engineered with a disulfide cross-link at residue 219, immediately preceding the self-association domain, show 6-fold binding enhancement to LC8 relative to the monovalent IC. In contrast, LC7, which binds IC at a site of a similar distance from LC8 as the disulfide cross-link, does not provide any discernable binding enhancement of IC to LC8. The gain from bivalency in this case is offset by the accompanying negative interactions associated with the loss of IC self-association within the 222–231 segment.

## LC8-promoted Swallow self-association

Genetic experiments on *Drosophila* ovaries show that the distribution of *bcd* mRNA during oogenesis and early embryogenesis depends on the interaction of LC8 with Swallow. Sequence analysis predicts a dimeric coiled-coil domain in the center of the 548 amino acid sequence, followed by a recognition sequence for LC8. A Swallow mutant lacking the coiled-coil domain and the LC8 recognition motif shows no localization of *bcd* mRNA [6]. Initial evidence for LC8-promoted structural changes was based on biophysical characterization of a Swallow construct containing the predicted coiled-coil domain and the LC8 site which shows that this domain is primarily monomeric at room temperature and that LC8 binding is required for its self-association and stability [10].

**Figure 3:**
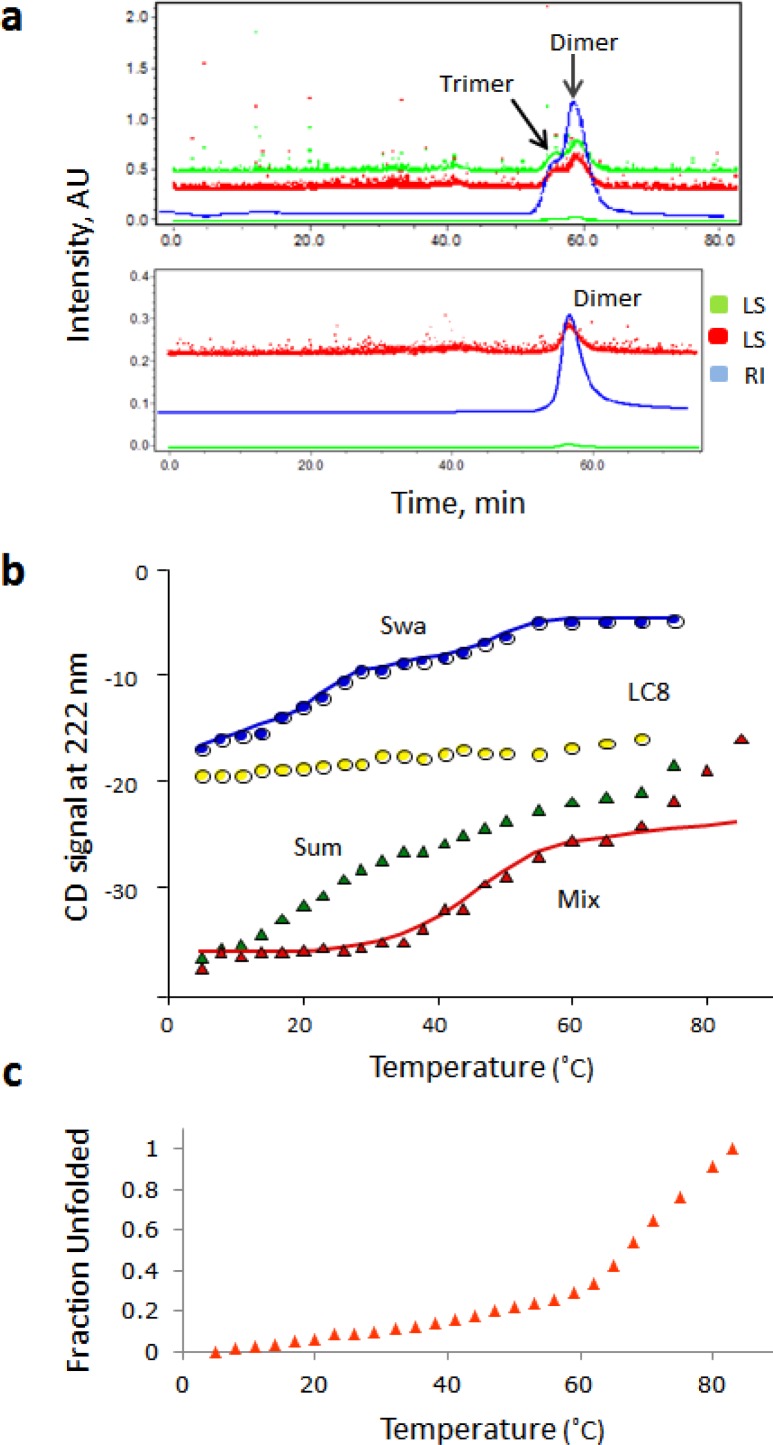
Association state and stability of Swallow dimer used as a model for the LC8-bound Swa_WT_. (a) Elution profiles of Swa_DIMER_ in different buffer conditions shown as overlays of the refractive index (RI) and light scattering at two different angles (red and green). At pH 8.0 in 20mM Tris buffer and 10 mM NaCl, the protein is about 70% dimer, 30% trimer (top), while at pH 5.6 in 20 mM MES and 10 mM NaCl, the protein is fully dimeric (bottom). The MALLS-determined molecular mass of Swa_DIMER_ is 20 kDa, consistent with the theoretical value of 18 kDa expected for a dimer. The mass determined for the trimer is 30 kDa. (b) Thermal denaturation curves of Swa_WT_ (blue), LC8 (yellow), their computed sum (green), and a 1:2 mixture of Swa/LC8 (red). The computed sum represents the hypothetical curve if there is no interaction (green curve, Sum). Swa_WT_ unfolding is multiphasic: The first step is protein concentration dependent and corresponds to dimer dissociation at less than 20 °C (blue Swa curve). In the Mix, the first transition is replaced with a plateau indicating that dimeric association is stabilized. (c) Thermal unfolding profiles of Swa_DIMER_ monitored at 222 nm showing that the protein does not start to unfold till after 60 °C. Figures b is adapted from [10].

Our LC8-promoted dimerization model is further supported by the following: CD data show that a Swallow construct corresponding to residues 206-297, Swa(206-297), is more stable when bound to LC8; the melting temperatures of free and LC8-bound Swa(206-297) are 15°C and 45°C, respectively (Figure 3). Swa(206-297) is more likely to covalently cross-link upon LC8 binding [10]. Coexpression of LC8 with Swa(206-297) significantly increases its expression and solubility suggesting binding is coupled to folding. More importantly, mutational design based on a hypothetical helical coiled-coil wheel, strongly supports formation of a coiled-coil by the observation that destabilizing and stabilizing mutations at the interface result in monomer (Swa_MONOMER_) or dimer (Swa_DIMER_), respectively [26].

For high resolution structural analyses of Swa_DIMER_, we tested its stability in different buffers at high protein concentrations. Ten buffer conditions were tested before identifying a buffer condition that gives only a dimer peak (Figure 3a). Swa_DIMER_ has similar CD-detected structure to the LC8-bound Swa_WT_ and similar unfolding profiles (Figure 3b and c), and therefore is a suitable model for the LC8-induced Swallow dimer [26]. Structural determination of Swa_DIMER_ involved both X-ray crystallography and NMR. Well diffracting crystals were obtained in the X-ray studies but the structure could not be solved by molecular replacement techniques due to the absence of homologous sequences in the protein data bank. Multi-wavelength anomalous dispersion phasing was also not possible due to aggregation of the protein upon incorporation of Se-Met into Swallow and the inability of finding buffer conditions that will eliminate aggregation, leaving out NMR as the only option for high resolution structural determination.

## NMR studies of the Swallow dimer

**Figure 4:**
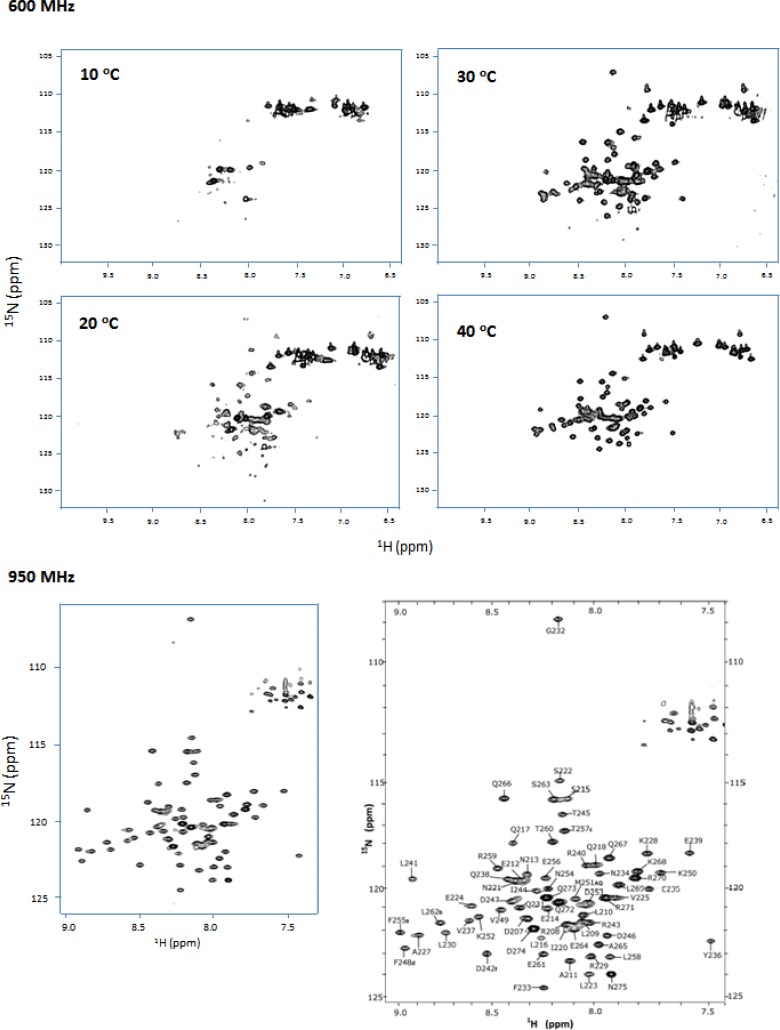
Effect of temperature and field strength on spectral quality. ^1^H-^15^N HSQC spectra collected at 600 MHz at increasing temperature in the range of 10-40 °C. Spectra collected at 40 °C and 950 MHz show significant improvement in resolution. Assignments spectrum collected at 950 MHz is adapted from [26].

The elongated structure of a coiled-coil protein in general results in fast transverse relaxation and subsequently broadened signals due to non-isotropic tumbling. For Swallow, which is predicted to form a coiled-coil, experimentation with different temperatures showed that at 40 °C, spectra were significantly improved due to faster tumbling and reduced relaxation rates. Furthermore, the limited resolution was also significantly improved by using a high field of 900 MHz that allowed determination of almost complete resonance assignments (Figure 4).

**Figure 5:**
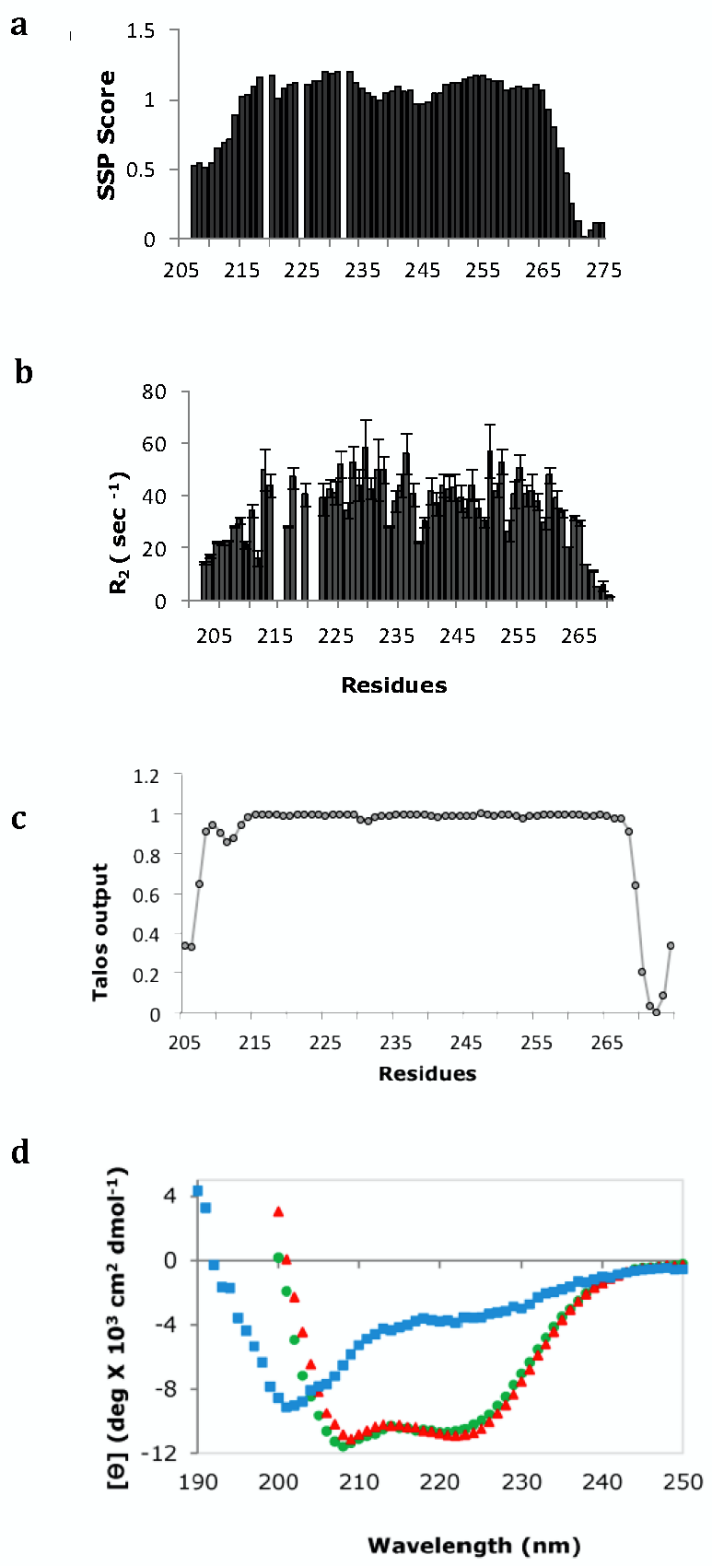
Secondary structure propensities and dynamics of Swa_DIMER_ (a) Secondary chemical shifts show high helical propensities as indicated in SSP scores per residue. (b) Plots of R_2_ showing different values across the chain indicating heterogeneous dynamics. (c) TALOS prediction shows high helical propensity across the chain and disorder at both termini. (d) Far-UV CD spectra of Swa_WT_ (green), and Swa_DIMER_ (red) showing a small increase in negative ellipticity at 208 nm for the dimer, consistent with supercoiling. Swa_MONOMER_ (blue) is predominantly unfolded. Figures a, b, and d are adapted from [26].

Secondary structure propensities were determined from the Cα, and Cβ chemical shifts using the programs SSP [27] and TALOS which in addition predicts *φ* and *ψ* torsion angle restraints [28] (Figure 5). The high helical propensity is confirmed by analysis of short and medium range NOEs that shows a long helix across the length of the chain. Dynamics data show that the protein is ordered except for few residues at both termini, and transverse relaxation rates R2 in particular, which report on conformational exchange in millisecond to microsecond time scale, show significant heterogeneity along the length of the chain suggesting some deviation from a standard coiled-coil structure (Figure 5) [26]. Circular dichroism spectroscopy differentiates between single and supercoiled helices on the basis of the ratio of ellipticity at 222 and 208 nm. While both Swa_DIMER_ and Swa_WT_ show double minima at 208 and 222 nm characteristic of an **α**–helical conformation, Swa_DIMER_ has a higher [**θ**222]/[**θ**208] ratio (close to 1), characteristic of supercoiling and further supporting the prediction of a coiled-coil structure (Figure 5d).

The challenge in solving a dimeric structure by NMR lies in the symmetrical nature of the dimer, which results in the same chemical shifts for the same proton from each chain. A single chemical shift for the same proton from each chain makes differentiating between intra monomer NOEs, those NOEs that are within each monomer, and inter monomer NOEs, the NOEs that are between the subunits across the dimer interface quite challenging. Since this is a coiled-coil, the interface is significantly long compared to globular dimers, and long-range NOEs are limited to those inter monomer NOEs across the interface. In dimeric coiled-coils, the major contributors to inter monomer NOEs are expected to involve residues at the *a* and *d* positions of the heptad repeats, as these positions are occupied by hydrophobic amino acids packed in a “ knob to holes” manner to form a supercoiled α -helix.

Since ^13^C and ^15^N edited NOESYs show both inter and intra monomer NOEs, to determine which of the NOEs are inter monomer NOEs only, a 3D ω1-^13^C/^15^N-filtered, ^13^C-separated NOESY-HSQC spectrum was collected on a sample that has one chain labeled with ^13^C and ^15^N and the other chain unlabeled. Sample preparation requires mixing equimolar amounts of ^13^C- and ^15^N-labeled and unlabeled protein in denaturing buffer to dissociate the dimer, and then followed by reconstitution by dialysis in renaturing buffer before protein concentration. The resulting sample is a mixture of 25% unlabeled/unlabeled, 25% labeled/labeled and 50% labeled/unlabeled. Data in this experiment will only be observed for the 50% labeled/unlabeled. Approximately 25 inter monomer NOEs were observed from which 13 were unambiguously assigned to those positions along the length of the predicted coiled coil, confirming that Swa_DIMER_ is indeed a coiled-coil [26]. A full structure awaits complete structural determination that requires assignment of a large number of inter and intra monomer NOEs.

## The process for LC8-promoted dimer formation

**Figure 6:**
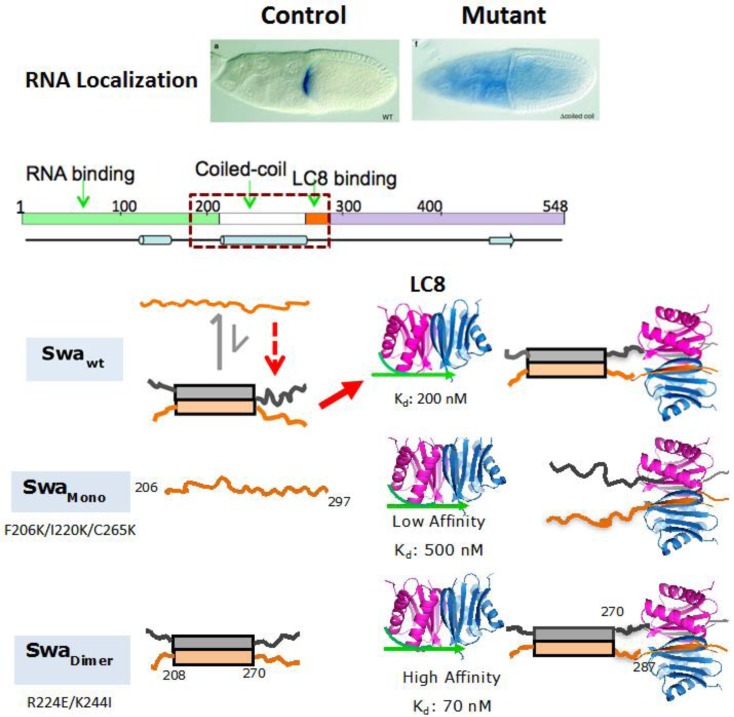
A model showing LC8 promoted Swallow dimerization. In the Swallow mutant egg chamber, bicoid mRNA fails to localize and is spread uniformly throughout the oocyte cytoplasm (blue). The Swallow mutant lacks the coiled-coil domain and the LC8 recognition motif. Full-length Swallow includes a putative RNA-binding domain at the N-terminus (green), a predicted α-helical coiled-coil region (residues 205–275) (white), and an LC8 recognition sequence (red). Predicted secondary structural elements [64] are shown as a cylinder for helix, arrow for strand and solid line for disorder****. Bars indicate the helical coiled-coil domain and lines indicate disorder. A construct of Swa_WT_ that included the coiled-coil and LC8 binding site (within dotted rectangle) is a mixture of high affinity dimer and a low affinity monomer. LC8 binding promotes dimer formation by binding to the dimeric low population and by mass action shifts the population of the bound to fully dimeric. LC8 and LC8/Swa structures are based on 3BRI and 3E2B pdb codes [8] and were generated using the program PyMOL [65]. Figure is adapted with modification from [26].

For both IC and Swallow, there is a minor population of a dimer which, as discussed below, binds LC8 significantly tighter than the major monomer population due to the bivalency effect and by mass action shifts the equilibrium to the dimer resulting in a complex that is a dimer (Figures 2 and 6). With the primarily monomeric IC, LC8 binds with an affinity of 9.8 µM. Self-associated bivalent IC, which is populated to varying extents in different species, is modeled in our studies with an engineered disulfide cross-link at residue 219 (81 residues apart from the LC8 recognition sequence). For the engineered 219C cross-linked dimer, LC8 binding affinity was enhanced 6-fold to 1.7 µM [24]. This increase in binding affinity is primarily contributed from the change in entropy (ΔH° of -14.0 to -14.3, and -TΔS° of 7.2 to 6.4 kcal/mol). Thus, observed increases in binding affinity apparently arise from entropic processes.

With Swallow, the self-associated form was engineered by introducing mutations that stabilize the coiled-coil rather than by disulfide cross-linking as with IC. Since Swallow is a mixture of monomer and dimer, for comparison of the energetics of binding between the dimer and monomer, we also produced the pure monomer by destabilizing the interface of the predicted coiled-coil. Both Swa_DIMER_ and Swa_MONOMER_ mutants contain the LC8 recognition site and the predicted self-association coiled-coil site. Both proteins are expected to adopt the same β-strand structure at the LC8/Swa interface, and to differ in their structure distant from the LC8 binding sequence. Swa_DIMER_ is a bivalent binding partner with two aligned recognition sequences for LC8, while Swa_MONOMER_ is a disordered monovalent chain with one LC8 recognition motif (Figure 6). A 17-residue linker separates the end of the coiled-coil (residue 270) [26] from the beginning of the LC8 site (residue 287) [1]. The dimer binds with an enhancement of 7-fold. As with IC, the enhancement is primarily of entropic origin (ΔΔG° of -1.1 kcal/mol, TΔΔS° of -1.2 kcal/mol, and ΔΔH and ΔΔCp of 0) [26] as expected from a bivalency effect [29]. A model showing LC8 interactions with Swa_MONOMER_, Swa_DIMER_ and Swa_WT_ (Figure 6) demonstrates that changes in solvent accessibility distant from the LC8-Swallow interface only occurs with Swa_WT_.

In summary, for both IC and Swallow, the common process that explains how LC8 binding promotes dimer formation is that LC8 binds the dimeric low population and by mass action shifts the population of the bound to fully dimeric.

## Summary and outlook

NMR is reviewed here as instrumental for characterization of proteins with high degree of disorder, high degree of heterogeneity, and that self-associate upon binding to LC8. A combination of proper constructs design, spectroscopy techniques such as circular dichroism to measure stability, fluorescence quenching to measure interactions at low protein concentrations are highly complementary to NMR especially for samples for which the large size and low concentration are necessary for their function. Thermodynamic measurements to elucidate entropic contributions, static light scattering to assess sample heterogeneity and innovative methods to probe self-association domains without solving a full structure, are collectively utilized in this work and underscore the importance of multidisciplinary approaches in solving complex biological problems.

The detailed work with IC and Swallow provide fundamental insight into the role of this highly conserved, ubiquitous and essential protein and its role in protein-protein interaction networks. LC8 interactions may be significant in regulating various cellular processes as LC8 itself can undergo regulatory switching. Phosphorylation of LC8 at Ser 88 is known to occur *in vivo* and results in abolishing or reducing binding to its partners [30, 31]. Phosphorylation of Ser 88 at the dimer interface results in dissociation of the LC8 dimer, and subsequent dissociation from dynein [30]. Binding is lost because the monomer lacks the groove that is necessary for binding [32, 33]. Dimerization is required for activity and phosphorylation can regulate this activity by acting as a rheostat to promote dissociation but still allows for tighter binding ligands to bind y shifting the equilibrium towards dimer [30].

IC and Swallow are emerging as elongated duplex proteins, containing a number of binding domains located at intervals along their length. We *distinguish two types: short consensus domains* that bind one or more proteins like LC8 and *dimerization domains* that bind the other duplex chain. We showed that LC8 binding promotes IC-IC or Swa-Swa dimerization domain binding (hence the dimerization hub hypothesis). Binding at any and/or all the domains leaves the rest of the N-terminal region of IC extended, disordered and presumably flexible, at least *in vitro*, with multiple attachment sites onto which other proteins assemble. This behavior appears to be common in assembly of protein complexes with high degree of intrinsic disorder. Similar work on other LC8 partners supports our interpretation of the LC8 role in general. With syntaphilin, LC8 stabilizes a helical coiled-coil domain within the microtubule binding region that could enhance syntaphilin-microtubule docking interactions [34]. With myosin V, LC8 promotes assembly of the coiled-coil domain [35–37]. With Nup159, LC8 may not be necessary for coiled-coil formation but five LC8 dimer molecules bind two chains of Nup159 at positions N-terminal to a predicted coiled-coil, while a large part of the protein remain disordered and accessible for interactions with other proteins [38]. We refer to IC, Swallow, and potentially Nup159 as Self-Interacting Tethering proteins, SIT, which self-associate and act as a tether with multiple attachment sites onto which other proteins assemble. While most partners are highly intrinsically disordered, such as Swallow, IC, and Nup159, other partners have high level of ordered structures such as Gephyrin and nNOS, which contain domains with known crystal structures but also have some stretches of disordered regions. With all these partners, however, LC8 binds within a protein segment that is predicted to have high level of disorder and therefore is anticipated to play the same role in ordering specific domains for subsequent interactions as it does for fully disordered proteins.
